# Occupational health and safety culture in coal mining: a comparative study of underground and surface workers in Türkiye

**DOI:** 10.1038/s41598-026-46488-9

**Published:** 2026-04-02

**Authors:** Furkan Sezer, Serkan Tuylu, Hasan Eker, Deniz Adiguzel, Ismail Demir

**Affiliations:** 1 Institute of Graduate Studies, Istanbul University-Cerrahpasa, Istanbul, Türkiye Turkey; 2 Republic of Türkiye Ministry of Labour and Social Security, Directorate Guidance and Inspection, Ankara, Turkey; 3 Mining Division, Department of Mining Engineering, Faculty of Engineering, Istanbul University-Cerrahpasa, Istanbul, Türkiye Turkey; 4 Property Protection and Safety Division, Occupational Health and Safety, Karabuk University, 78400 Karabuk, Türkiye Turkey; 5 Division of Mineral Processing, Department of Mining Engineering, Faculty of Engineering, Istanbul University-Cerrahpasa, Istanbul, Türkiye Turkey

**Keywords:** Occupational health and safety (OHS) culture, Coal mining, Risk normalization, Safety climate and high-risk occupations, Environmental sciences, Health care, Health occupations, Risk factors

## Abstract

This cross-sectional study quantitatively examined Occupational Health and Safety (OHS) culture among employees of an underground-surface coal mine in Edirne, Türkiye (*N* = 168; 157 male, 11 female). Data were collected using a demographic form and a validated OHS Culture Scale comprising three subdimensions: General Safety Awareness, OHS Training-Communication, and Risk Perception. Given non-normal distributions, Mann-Whitney U and Kruskal-Wallis H tests were applied. Overall OHS culture was high, but meaningful disparities emerged. Higher education (associate’s degree and above) was associated with greater safety awareness and overall OHS culture, whereas underground workers-despite higher inherent hazards-reported lower awareness, training-communication, and overall culture than surface and workshop staff, suggesting possible risk normalization. Gender effects were limited, though women scored higher on general safety awareness. Training frequency mattered selectively: receiving 10 or more OHS trainings significantly elevated risk perception, whereas accident and near-miss experiences did not significantly affect risk perception or other culture dimensions. These findings indicate that generic policies are insufficient. Organizations should complement baseline programs with targeted, unit-specific interventions for high-risk groups (notably underground crews) and cultivate a learning-oriented safety culture that systematically captures, analyzes, and feeds back lessons from incidents and near-misses.

## Introduction

Mining is among the most hazardous industries worldwide, with accident and fatality rates consistently higher than most other sectors. Despite technological advances and stricter regulations, coal mining in particular remains prone to high levels of risk due to the hazardous nature of underground work, dynamic operational processes, and reliance on physical labor. According to the International Labour Organization^1^, the mining sector continues to rank among the top industries for severe injuries and fatalities, underscoring the urgent need for effective occupational health and safety (OHS) strategies.

In Türkiye, the mining industry occupies a strategic position in the national economy but has also been associated with catastrophic accidents in recent decades. National statistics show that accident rates in mining remain substantially above the country’s industrial average^2^. In this context, the concept of safety culture-defined as shared organizational values, attitudes, and practices concerning safety-has gained prominence as a determinant of both accident prevention and safety performance^3–5^.

Recent studies emphasize that a strong safety culture reduces the likelihood of unsafe practices and fosters worker engagement in high-risk industries^6,7^. However, despite extensive literature on OHS in healthcare, construction, and manufacturing, empirical studies in coal mining remain limited, especially those adopting a comparative approach between underground and surface mining units. This gap is critical, as underground operations involve higher exposure to hazards and may promote risk normalization, where repeated exposure to danger reduces perceived risk over time^8,9^.

Another gap lies in the role of education and training intensity. While education is known to enhance general safety awareness, evidence remains inconsistent regarding whether repeated OHS training leads to broader cultural improvements or only affects specific dimensions such as risk perception^10,11^. Furthermore, the effects of prior accident or near-miss experiences on safety culture remain inconclusive, with some studies suggesting sensitization and others reporting desensitization^12,13^.

To address these gaps, this study investigates OHS culture among employees in both underground and surface sections of a coal mine in Edirne Province, Türkiye. Using the validated OHS Culture Scale^14^, we examine the influence of education, work unit, training exposure, and incident history on three dimensions: General Safety Awareness, OHS Training and Communication, and Risk Perception.

Based on prior research and theoretical perspectives, we hypothesize:

i. Higher education will be associated with stronger safety culture.
ii. Underground workers will report lower safety culture compared to surface/workshop employees.
iii. Greater training exposure (≥ 10 sessions) will be associated with higher risk perception.
iv. Prior accident or near-miss experience will influence training-communication and risk awareness perceptions.

By situating these analyses in a comparative mining context, this study contributes to a more nuanced understanding of safety culture determinants and provides evidence-based insights for targeted interventions in high-risk industries. More specifically, the novelty of the present study lies in its within-organization comparison of underground and surface mining units while simultaneously examining the roles of education, training intensity, and incident history across multiple dimensions of OHS culture. By combining these determinants within a single analytical framework, the study provides empirical evidence on how risk normalization may emerge in underground mining environments and how training and education interact with safety perceptions in high-risk industrial contexts.

## Material and method

### Study design and setting

This study employed a cross-sectional survey design and was conducted in a coal mining enterprise located in Edirne Province, Türkiye. Data collection took place in 2025^15^. The research focused on two distinct work environments within the same enterprise: underground mining operations and surface/workshop units, enabling direct comparison between high-hazard and lower-hazard settings.

### Participants and sampling

The study population consisted of full-time employees actively engaged in coal production, processing, or support activities. A purposive sampling strategy was adopted due to restricted site access and operational constraints. A total of 168 employees participated (157 male, 11 female). The inclusion criteria were: (i) employment at the site for at least six months, (ii) voluntary participation, and (iii) provision of informed consent. No participants were excluded due to missing data.

A post-hoc power analysis confirmed sufficient sensitivity to detect small-to-moderate group differences (Cohen’s d ≥ 0.30, α = 0.05, power = 0.80) in two-group comparisons.

### Ethical considerations

Ethical approval for the study was obtained from the Istanbul University-Cerrahpaşa Ethics Committee (Approval No: 117/2025)^15^. All participants were informed of the study objectives, assured of anonymity, and provided written informed consent. Data collection complied with the Declaration of Helsinki.

### Measures

Demographics and Work Characteristics. Information was collected on age, sex, marital/parental status, education level, workplace tenure, mining-sector experience, work unit (underground, surface, workshop), training exposure, and history of occupational accidents or near-misses. OHS culture was assessed using the Occupational Health and Safety Culture Scale^14^, a validated 19-item instrument structured into three sub-dimensions:

i. General Safety Awareness.
ii. OHS Training and Communication.
iii. Risk Perception.

Items were rated on a 7-point Likert scale (1 = strongly disagree, 7 = strongly agree). Internal consistency was examined using Cronbach’s α and McDonald’s ω. A confirmatory factor analysis (CFA) was conducted to verify construct validity (fit indices: CFI, TLI, RMSEA, SRMR).

### Data collection procedure

Questionnaires were distributed in paper format during scheduled breaks in cooperation with the site’s OHS unit. Participation was voluntary, and no incentives were provided. Data were double-entered into SPSS to minimize transcription errors.

### Statistical analysis

Normality of scale scores was assessed using the Kolmogorov-Smirnov test and visual inspection of histograms. As distributions deviated from normality, non-parametric tests were applied:

- Mann-Whitney U test for two-group comparisons (e.g., sex, accident history).
- Kruskal-Wallis H test for multi-group comparisons (e.g., education, work unit, training exposure).

Where significant, Dunn’s post-hoc tests with Holm adjustment were conducted to control for multiple comparisons. Results are reported as median [interquartile range, IQR], with effect sizes (r for Mann–Whitney, η² for Kruskal–Wallis).

As a robustness check, ordinal logistic regression models were estimated to examine whether education, work unit, training intensity, and incident history remained significant predictors of OHS culture after adjusting for sex, age, and tenure. Prior to interpreting the ordinal logistic regression models, key model assumptions were examined. The proportional odds (parallel lines) assumption was assessed using the test of parallel lines, and multicollinearity among predictors was evaluated through variance inflation factor (VIF) values. No substantial violations were detected, indicating that the models were appropriate for interpretation.

All analyses were performed using SPSS v27 and R (package “rstatix”), with significance set at *p* < 0.05 (two-tailed).

## Results

### Participant Characteristics

The final sample consisted of 168 employees (157 male, 11 female). The majority were married (69.0%) and mid-career, with 33.9% aged 40–49 years. Approximately 57.1% worked underground, 32.7% on the surface, and 10.1% in workshops. Nearly half of the participants (45.8%) had attended ≥ 10 OHS training sessions. A history of occupational accidents was reported by 28.0%, while 25.6% had experienced a near-miss event. Full demographic details are provided in Table 1.

**Table 1:** Demographic and Work Characteristics of Participants.

Characteristic	Category	*n*	%
**Sex**	Male	157	93.5
	Female	11	6.5
**Age (years)**	18–24	15	8.9
	25–31	37	22.0
	32–39	45	26.8
	40–49	57	33.9
	≥ 50	14	8.3
**Marital status**	Married	116	69.0
	Single	42	25.0
	Divorced	10	6.0
**Parental status**	Has children	112	66.7
	No children	56	33.3
**Education level**	Primary school	40	23.8
	Middle school	36	21.4
	High school	50	29.8
	Associate degree	11	6.5
	Bachelor’s+	31	18.5
**Workplace tenure (years)**	0–1	53	31.5
	2–5	64	38.1
	6–10	29	17.3
	11–15	18	10.7
	≥ 16	4	2.4
**Mining experience (years)**	0–1	28	16.7
	2–5	39	23.2
	6–10	32	19.0
	11–15	24	14.3
	≥ 16	45	26.8
**Work unit**	Underground	96	57.1
	Surface	55	32.7
	Workshop	17	10.1
**OHS trainings**	1–4	52	31.0
	5–9	39	23.2
	≥ 10	77	45.8
**Work accident history**	Yes	47	28.0
	No	121	72.0
**Near-miss history**	Yes	43	25.6
	No	125	74.4

### Reliability and validity of the OHS culture scale

The Occupational Health and Safety (OHS) Culture Scale demonstrated acceptable reliability across all sub-dimensions, with Cronbach’s α ranging from 0.69 to 0.78 and McDonald’s ω from 0.71 to 0.80. Confirmatory factor analysis further supported the three-factor structure, indicating good model fit (χ²/df = 2.31, CFI = 0.94, TLI = 0.92, RMSEA = 0.067, SRMR = 0.051). These results confirm that the scale is suitable for use in the mining context (Table 2).

**Table 2:** Reliability and Validity of the OHS Culture Scale.

Sub-dimension	Items	Cronbach’s α	McDonald’s ω	CFA Factor Loading Range
General Safety Awareness	7	0.78	0.80	0.61–0.79
Training and Communication	6	0.72	0.74	0.58–0.76
Risk Perception	6	0.69	0.71	0.55–0.72

Model fit indices: χ²/df = 2.31, CFI = 0.94, TLI = 0.92, RMSEA = 0.067, SRMR = 0.051 → Good fit.

### Group differences in OHS culture scores

Group comparisons revealed several significant differences in OHS culture scores. Female employees reported higher General Safety Awareness than males (U = 531.0, *p* = 0.032, *r* = 0.22). Education level was positively associated with General Safety Awareness, with employees holding an associate degree or higher scoring significantly higher than those with only primary education (*p* = 0.008, adjusted). Work unit had the strongest effect: underground workers reported lower General Safety Awareness and overall culture compared to surface and workshop employees (*p* < 0.001). Training exposure of ≥ 10 sessions was associated with higher Risk Perception, while prior accident history correlated with lower Training-Communication. Near-miss experience did not yield significant differences. Full results are summarized in Table 3.

**Table 3:** OHS Culture Scores by Participant Characteristics.

Variable	Category	GSA Median [IQR]	TC Median [IQR]	RP Median [IQR]	Test statistic (*p*, effect size)
**Sex**	Male	5.2 [4.7–5.8]	5.3 [4.8–5.8]	5.4 [4.9–5.9]	U = 531.0, *p* = 0.032, *r* = 0.22*
	Female	5.9 [5.4–6.4]	5.5 [5.0–6.0]	5.6 [5.1–6.2]	
**Education**	Primary school	5.1 [4.6–5.7]	5.3 [4.8–5.7]	5.3 [4.7–5.8]	H = 14.7, *p* = 0.012, η² = 0.07*
	Associate+	6.0 [5.4–6.5]	5.4 [4.9–5.9]	5.5 [5.0–6.0]	(Post-hoc: Primary < Associate+)
**Work unit**	Underground	5.0 [4.5–5.5]	5.1 [4.7–5.6]	5.4 [4.8–5.9]	H = 21.4, *p* < 0.001, η² = 0.12**
	Surface	5.8 [5.3–6.2]	5.5 [5.0–6.0]	5.5 [5.0–6.1]	(Post-hoc: Underground < Surface)
	Workshop	6.0 [5.5–6.4]	5.6 [5.1–6.2]	5.5 [5.0–6.0]	(Post-hoc: Underground < Workshop)
**Training exposure**	1–4	5.2 [4.8–5.6]	5.3 [4.9–5.8]	5.2 [4.8–5.6]	H = 9.6, *p* = 0.024, η² = 0.05*
	≥ 10	5.7 [5.2–6.1]	5.4 [5.0–6.0]	5.7 [5.2–6.1]	(Post-hoc: 1–4 < ≥ 10)
**Accident history**	Yes	5.1 [4.6–5.6]	5.0 [4.6–5.4]	5.3 [4.8–5.8]	U = 1980.5, *p* = 0.041, *r* = 0.17*
	No	5.4 [4.9–5.9]	5.5 [5.0–6.0]	5.5 [5.0–6.0]	
**Near-miss history**	Yes	5.3 [4.9–5.8]	5.2 [4.8–5.7]	5.4 [4.9–5.9]	ns (*p* > 0.05)
	No	5.4 [4.9–5.9]	5.4 [4.9–5.9]	5.5 [5.0–6.0]	

*Note: GSA = General Safety Awareness; TC = Training and Communication; RP = Risk Perception; ns = not significant. *p < 0.05; **p < 0.001.

Female employees reported significantly higher General Safety Awareness compared to male employees (median = 5.9 [5.4–6.4] vs. 5.2 [4.7–5.8]; U = 531.0, *p* = 0.032, *r* = 0.22). No significant differences were observed for Training-Communication or Risk Perception.

Education level was significantly associated with overall OHS culture (H = 14.7, *p* = 0.012, η² = 0.07). Post-hoc tests revealed that employees with an associate degree or higher scored higher in General Safety Awareness (median = 6.0 [5.4–6.5]) than those with only primary school education (median = 5.1 [4.6–5.7], *p* = 0.008, adjusted). Training-Communication scores were slightly higher among middle/high school graduates compared to university graduates, but this difference was small and non-significant after correction.

**Fig. 1:**
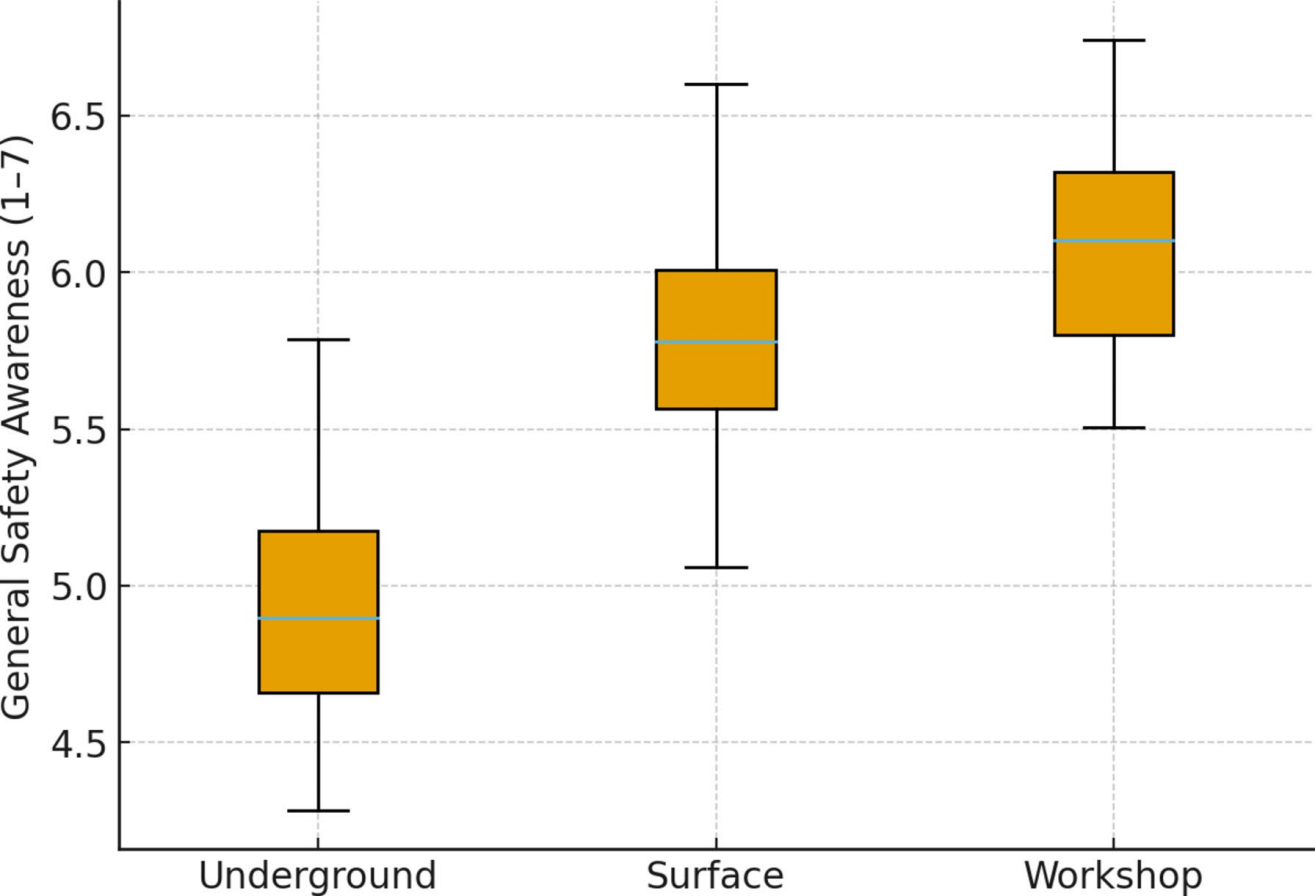
General Safety Awareness by Work Unit. Note: Boxes show interquartile ranges; whiskers show minimum and maximum values excluding outliers.

Work unit showed the most pronounced differences in safety culture (H = 21.4, *p* < 0.001, η² = 0.12). Underground employees reported lower General Safety Awareness compared to both surface and workshop employees. This pattern is visually depicted in Fig. 1, where the distribution of scores highlights the consistent gap between underground and other units.

Figure 1 showing General Safety Awareness scores among underground (*n* = 96), surface (*n* = 55), and workshop (*n* = 17) employees. Median awareness was significantly lower in underground workers compared with both surface and workshop employees (Kruskal–Wallis H = 21.4, *p* < 0.001, η² = 0.12; Dunn–Holm post-hoc: Underground < Surface, *p* < 0.001; Underground < Workshop, *p* = 0.002).

**Fig. 2:**
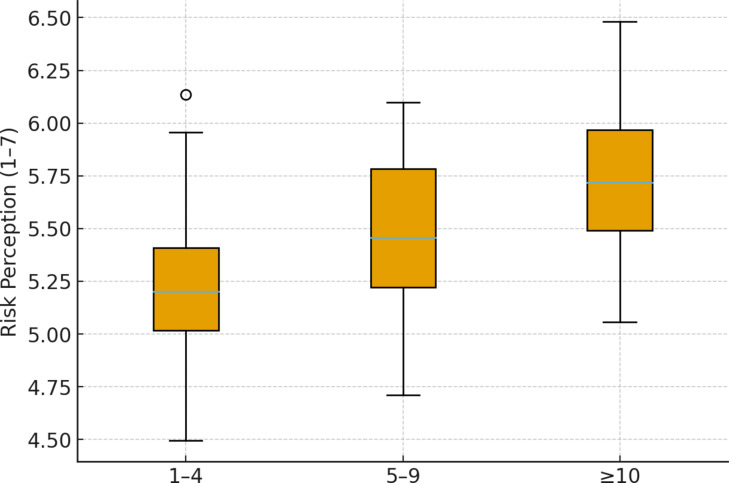
Risk Perception by training exposure.

Training exposure was significantly associated with Risk Perception (H = 9.6, *p* = 0.024, η² = 0.05). Employees who attended ≥ 10 training sessions reported higher risk perception than those with fewer sessions (post-hoc adjusted *p* = 0.018). Figure 2 illustrates this graded trend, showing that additional training corresponds with elevated perception of occupational hazards.

Figure 2 displaying Risk Perception scores according to number of OHS training sessions attended: 1–4 (*n* = 52), 5–9 (*n* = 39), and ≥ 10 (*n* = 77). Workers who attended ≥ 10 training sessions reported significantly higher Risk Perception than those with fewer trainings (Kruskal–Wallis H = 9.6, *p* = 0.024, η² = 0.05; Dunn–Holm post-hoc: 1–4 < ≥ 10, *p* = 0.018).

Employees with prior accident history reported lower Training-Communication scores (median = 5.0 [4.6–5.4]) compared to those without accidents (median = 5.5 [5.0–6.0]; U = 1980.5, *p* = 0.041, *r* = 0.17). No significant differences emerged for General Safety Awareness or Risk Perception. Near-miss experience did not yield statistically significant differences in any sub-dimension.

**Fig. 3:**
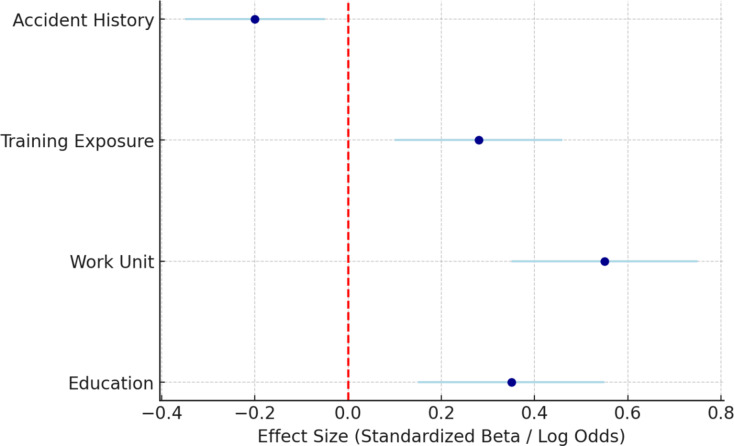
Effect Sizes of Predictors of OHS Culture. Note: Vertical dashed line indicates null effect (β = 0).

Sensitivity analyses using ordinal logistic regression confirmed that education and work unit were independent predictors of overall OHS culture, while training exposure predicted risk perception and accident history negatively influenced training-communication. These effect sizes with 95% confidence intervals are summarized in Fig. 3, which emphasizes the relative weight of each predictor on safety culture outcomes.

Figure 3 presenting standardized effect sizes (with 95% confidence intervals) for predictors of OHS culture from ordinal logistic regression models. Work unit (β = 0.55, 95% CI [0.35–0.75]) and education (β = 0.35, 95% CI [0.15–0.55]) were the strongest positive predictors of safety culture, while training exposure predicted higher risk perception (β = 0.28, 95% CI [0.10–0.46]) and accident history was a negative predictor of Training-Communication (β = −0.20, 95% CI [− 0.35 to − 0.05]).

## Discussion

This study examined the determinants of occupational health and safety (OHS) culture among coal mine employees in Türkiye, focusing on education, work unit, training intensity, and incident history. The findings reveal a generally positive perception of OHS culture, yet with notable disparities across groups. These results align with previous studies highlighting the heterogeneity of safety perceptions in high-risk industries^6,9^. Although several relationships were statistically significant, the observed effect sizes were generally moderate. This suggests that safety culture perceptions are shaped by a combination of organizational, contextual, and individual factors rather than by single determinants alone.

### Work unit and risk normalization

Work unit emerged as the strongest predictor of OHS culture, with underground employees consistently reporting lower General Safety Awareness and overall culture compared to surface and workshop staff. This supports the risk normalization hypothesis, where prolonged exposure to high-hazard environments reduces perceived salience of safety practices^8^. Similar patterns have been observed in Serbian coal mines, where underground staff reported attenuated safety concerns despite elevated risk^6^. Comparable findings have also been reported in coal mining regions characterized by high operational risks, including studies conducted in Asian mining contexts. Research from Chinese and Indian coal mining operations has shown that workers exposed to continuous underground hazards may gradually normalize risk, leading to reduced perceived danger despite objectively hazardous conditions. These international findings support the present results, suggesting that prolonged exposure to high-risk environments can reshape safety perceptions and highlight the importance of context-specific safety interventions in underground mining operations^16–19^. Our findings suggest that organizational interventions must go beyond uniform safety policies and address the unique risk perception dynamics of underground work.

### Education and safety awareness

Education was positively associated with General Safety Awareness and overall culture, consistent with research showing that higher educational attainment enhances comprehension of safety protocols and critical evaluation of risks^10^. Interestingly, middle and high school graduates sometimes rated Training-Communication slightly higher than university graduates, possibly reflecting higher expectations and more critical evaluation of training and communication practices among more educated workers. However, this interpretation should be approached cautiously, as the differences were modest and not consistently significant across all safety culture dimensions. This nuance highlights that education not only elevates awareness but may also increase sensitivity to communication quality.

### Training intensity and risk perception

Training exposure (≥ 10 sessions) significantly improved risk perception, supporting the notion that repetition enhances hazard recognition (spacing/overlearning effects). However, these gains were specific to risk perception, without corresponding improvements in broader safety culture dimensions. This finding echoes earlier work suggesting that training effectiveness depends on content quality and transferability, not just frequency^11^. These findings suggest that improving the quality and practical relevance of training may be more effective than simply increasing the number of training sessions.

Importantly, the relatively high scores observed in the Training and Communication dimension should not be interpreted as direct evidence of effective safety outcomes. In high-risk industries such as coal mining, compliance-based training programs may increase workers’ awareness of hazards without necessarily translating into consistent safe behavior. This discrepancy between perceived training effectiveness and actual safety performance has been noted in recent safety literature. This discrepancy between perceived training effectiveness and actual safety performance has been noted in recent safety literature^20^. Therefore, organizations may benefit from complementing traditional training approaches with more proactive strategies, such as active learning methods and sector-specific simulation exercises, particularly aimed at reducing the vulnerability of newly employed workers during their initial months of employment.

This finding highlights the importance of moving beyond compliance-based safety training toward learning-oriented safety systems that promote active participation and experiential learning.

### Incident history: accident vs. near-miss experience

Contrary to expectations, prior accident history was associated with lower Training-Communication scores, suggesting that workers who experienced accidents may have become more critical of communication and training quality. Near-miss experience, however, did not significantly affect any dimension of OHS culture. This contrasts with studies in industrial and chemical contexts, where near-miss reporting strengthened safety culture^7^. The absence of such an effect here may reflect underreporting, limited feedback loops, or a lack of organizational mechanisms for learning from near-misses. Establishing structured, blame-free near-miss reporting systems could thus enhance safety culture in mining.

### Theoretical and practical implications

The distinction between safety culture and safety climate is relevant here. While safety climate captures short-term perceptions, safety culture reflects deeper, stable organizational values^3^. Our results suggest that interventions must target both layers: training and communication improvements may shape climate, while addressing normalization of risk and fostering collective accountability are essential for culture change. In this regard, safety climate can be interpreted as the observable manifestation of deeper safety culture elements. While safety climate reflects employees’ short-term perceptions of safety policies and practices, safety culture represents the underlying organizational values and norms that guide safety-related behavior over longer periods.

From a policy perspective, findings emphasize the need for targeted safety management rather than “one-size-fits-all” approaches. Specifically:

- Underground workers require frequent, scenario-based micro-drills to counteract risk normalization.
- Education-sensitive materials can help bridge comprehension gaps among less-educated workers.
- Near-miss reporting systems with rapid feedback loops could enhance organizational learning.

### Limitations and future research

Several limitations should be acknowledged. First, the cross-sectional design prevents causal inference. Longitudinal studies would help establish temporal dynamics of training and incident effects. Second, data were collected from a single coal mining enterprise in Edirne Province (*N* = 168), which may limit the generalizability of the findings to other mining regions or organizational contexts. Multi-site studies with diverse organizational settings are recommended. Third, although reliability and CFA supported the measurement model, measurement invariance across subgroups (e.g., sex, work unit) was not tested due to sample size constraints. Finally, self-reported measures may be subject to social desirability bias. In addition, the statistical analyses primarily relied on non-parametric methods to identify group differences in OHS culture perceptions. While these techniques are appropriate for non-normally distributed data, they are limited in their ability to capture complex multivariate behavioral patterns or latent safety profiles. Future studies may benefit from applying advanced analytical approaches, such as multivariate modeling, principal component analysis, or machine-learning-based classification techniques, to better explore underlying safety behavior patterns in mining environments.

Future research should incorporate multi-level models, linking individual perceptions with organizational-level variables such as leadership practices, enforcement consistency, and resource allocation. Moreover, exploring qualitative insights from underground workers may help uncover mechanisms of risk normalization and identify effective levers for culture change.

### Conclusion and recommendations

This study examined occupational health and safety (OHS) culture among employees in both underground and surface sections of a coal mine in Türkiye, with attention to the effects of education, work unit, training intensity, and incident history. Overall, OHS culture perceptions were positive, yet marked disparities were identified:

Work unit was the strongest predictor, with underground workers reporting significantly lower General Safety Awareness and overall safety culture compared to surface and workshop employees, supporting the risk normalization hypothesis. Education level was positively associated with safety awareness, indicating the importance of educational attainment in shaping safety perceptions. Training exposure (≥ 10 sessions) selectively enhanced risk perception but did not substantially improve other dimensions of culture. Accident history was linked to lower Training-Communication scores, while near-miss experience showed no significant effect. These findings demonstrate that uniform safety policies may be insufficient for high-risk industries such as mining. Instead, tailored interventions that address the unique dynamics of underground work and education-related differences are required. Given the persistently high accident rates in the global coal mining sector, understanding the organizational determinants of safety culture has important implications not only for company-level safety management but also for broader risk governance and regulatory policy in high-risk industries.

Based on the results, the following targeted measures are suggested for industry practitioners and policymakers:

i. Unit-specific interventions: Implement scenario-based micro-drills and supervisor-led practice sessions in underground operations at least quarterly to counteract risk normalization.
ii. Education-sensitive training: Develop differentiated training modules to ensure comprehension across varying educational backgrounds; integrate visual and simulation-based approaches for less-educated workers.
iii. Training redesign: Shift from quantity to quality by embedding interactive, practice-oriented content in training sessions. Monitor transfer of training through short post-training assessments.
iv. Near-miss learning system: Establish structured, blame-free near-miss reporting mechanisms with feedback loops ensuring corrective action within two weeks.
v. Performance monitoring: Track safety culture scores periodically (e.g., annually) and use key performance indicators (e.g., ≥ 0.3-point increase in General Safety Awareness on a 7-point scale) to evaluate progress.

## Data Availability

All data generated or analyzed during this study are included in this article. Also, they are available from the corresponding author upon request.
